# ADAM-10 Regulates MMP-12 during Lipopolysaccharide-Induced Inflammatory Response in Macrophages

**DOI:** 10.1155/2022/3012218

**Published:** 2022-09-16

**Authors:** Yan Jiang, Qiming Gong, Jinmei Huang, Yuanxun Gong, Qiang Tang, Dalong Wei, Qianli Tang, Jingjie Zhao, Jian Song, Lingzhang Meng

**Affiliations:** ^1^Medical College, Guangxi University, Nanning, Guangxi Zhuang Autonomous Region, China; ^2^West Guangxi Key Laboratory for Prevention and Treatment of High-Incidence Diseases, Youjiang Medical University for Nationalities, Baise, Guangxi Zhuang Autonomous Region, China; ^3^Department of Nephrology, The Affiliated Hospital of Youjiang Medical University for Nationalities, Baise, Guangxi Zhuang Autonomous Region, China; ^4^Guangxi University of Chinese Medicine, Nanning, Guangxi Zhuang Autonomous Region, China; ^5^Life Science and Clinical Research Center, The Affiliated Hospital of Youjiang Medical University for Nationalities, Baise, Guangxi Zhuang Autonomous Region, China; ^6^Burn Plastic & Trauma Surgery Department, The Affiliated Hospital of Youjiang Medical University for Nationalities, Baise, Guangxi Zhuang Autonomous Region, China; ^7^Center for Systemic Inflammation Research (CSIR), School of Preclinical Medicine, Youjiang Medical University for Nationalities, Baise, Guangxi Zhuang Autonomous Region, China; ^8^Institute of Cardiovascular Sciences, Guangxi Academy of Medical Sciences, Nanning, Guangxi Zhuang Autonomous Region, China

## Abstract

A disintegrin and metalloprotease 10 (ADAM-10), a member of the ADAM protease family, has biological activities related to TNF-*α* activation, cell adhesion, and migration, among other functions. Macrophages are important immune cells that are involved in the inflammatory response of the body. ADAM-10 is involved in inflammatory responses, but the specific regulatory mechanisms are not fully understood. In this study, we investigated the regulatory mechanism of ADAM-10 in the lipopolysaccharide-promoted proliferation (LPS) of the macrophage inflammatory response. Differentially expressed or regulated proteins were identified in interfered ADAM-10 (sh ADAM-10) macrophages using tandem mass tag (TMT) proteomics. The changes and regulatory role of ADAM-10 during LPS-induced inflammatory response in normal, interfering, and overexpressing ADAM-10 (EX ADAM-10) cells were determined. Results indicated that ADAM-10 interference affected inflammation-related pathways and reduced matrix metalloproteinase 12 (MMP-12) protein levels, as identified by TMT proteomics. In normal cells, LPS decreased ADAM-10 gene expression, but promoted ADAM-10 secretion, *MMP-12* and *TNF-α* gene expression, and MMP-12, iNOS, IL-10, and cyclinD1 protein expression. Additionally, ADAM-10 knockdown decreased macrophage viability in sh-ADAM-10 cells. Moreover, an MMP-12 inhibitor had no impact on the viability effect of LPS on cells or the expression of *ADAM-10*. iNOS expression decreased, whereas IL-10 expression increased after ADAM-10 depletion. ADAM-10 knockdown decreased *MMP-12*, *iNOS*, *TNF-α*, *IL-1β*, and *FKN*, while overexpression had an opposite effect. ADAM-10 overexpression further increased *MMP-12*, *iNOS*, and *TNF-α* gene expression in response to LPS. Cell viability was increased in EX ADAM-10 cells, and ADAM-10 secretion was further increased in the EX and LPS groups. Flow cytometry and immunofluorescence staining revealed that EX-ADAM 10 cells had increased iNOS expression, which acted as an IL-6 expression driver. In summary, we found that ADAM-10 is activated by LPS and positively participates in LPS-stimulated macrophage inflammatory responses by positively regulating MMP-12 during the inflammatory process.

## 1. Introduction

Macrophages play an important role in immune defense in all multicellular organisms [1], as well as a key regulatory role in immune homeostasis [2], and participate in various immune responses, such as inflammatory responses, wound healing, and tumor progression [3, 4].

A disintegrin and metalloprotease (ADAM) proteins are a family of membrane-anchored cell surface proteases structurally associated with regeneration [5]. ADAM proteins play two biological roles: (1) as membrane-anchored metalloproteinases associated with cell surface protein shedding [6] and (2) as soluble proteases (known as exonucleases) involved in cleaving different cell surface protein matrix molecules (e.g., receptors, growth factors, cytokines, and matrix metalloproteinases) [7].

ADAM-10, a member of the ADAM family, activates the cleavage of TNF-*α* [8, 9] and exerts a regulatory effect on cell growth, adhesion, migration, and spinal stability [10–13]. ADAM-10 promotes inflammatory cytokine production through ERK1/2-mediated activation of NF-*κ*B in macrophages [14]. However, the specific regulatory mechanisms of ADAM-10 in LPS-induced inflammatory response in macrophages remain unclear.

In this study, we used lipopolysaccharide (LPS) to induce inflammation in macrophages and explored the regulatory role of ADAM-10 in inflammatory responses. Our results showed that LPS induced inflammatory responses in macrophages via ADAM-10, and that the regulatory role of ADAM-10 was positively associated with matrix metalloproteinase 12 (MMP-12).

## 2. Materials and Methods

### 2.1. Cell Cultures and Treatments

RAW264.7 and J774a.1, which are typical macrophage cell lines, are widely used in immunology and cell biology research [15]. RAW264.7 cells were purchased from the Conservation Genetics CAS Kunming Cell Bank (Kunming, China). J774a.1 cells were obtained from the Beina Chuanglian Biology Research Institute (Beijing, China). Both cell lines were cultured in Dulbecco's modified Eagle medium (DMEM) (Gibco) with 12% fetal bovine serum (South America) at 37°C and 5% CO_2_. Cells were transfected using LV-Adam10-RNAi to construct the sh ADAM-10 cell line according to the manufacturer's instructions (Shanghai GeneChem Co., Shanghai, China). Cells were also transfected with an EX ADAM-10 plasmid (Shanghai Genechem Co., Shanghai, China) to overexpress ADAM-10. Stable cells were grouped as follows: (1) the control (CON) group; (2) LPS group, 1 *μ*g/ml of LPS (L2880, Sigma) administered to cells for 12 h; and (3) MMP-12 inhibitor+LPS group (MMP-12 inh+LPS), 1.5 *μ*M of MMP-12 inhibitor (PF-00356231, MCE, Shanghai, China) for 12 h, followed by the addition of 1 *μ*g/ml of LPS for 12 h.

### 2.2. sh ADAM-10 and EX ADAM-10 Stable Cell Establishment

A sh ADAM-10 stable cell line was transfected with LV-Adam10-RNAi for 14 h, the medium was changed, and the cells were observed after 72 h using a fluorescence microscope (Olympus, Tokyo). A stable cell line was formed by puromycin screening using 5 *μ*g/ml puromycin for 24 h. EX ADAM-10 cells were established using EX ADAM-10 plasmid transfection for 24 h with lipo8000 (Beyotime, Shanghai, China), and the cells were subsequently grouped for experiments.

### 2.3. Proteomic Analysis

Stable normal and sh ADAM-10 cells were lysed in SDT buffer. Cellular proteins were assayed using TMT-labeled proteomics (CSC New Life, Shanghai) to identify differentially expressed proteins between the two cell lines.

### 2.4. Cell Viability Assays

The effect of different stimulations on cell viability was determined using a cell counting kit-8 (CCK-8, 40203ES76, YESEN, Shanghai, China) according to the manufacturer's instructions. Cells (8 × 10^3^) were seeded in 96-well plates for 12 h, after cells were stimulated as described above. Following treatment, 5 *μ*l of the CCK-8 solution was added and incubated at 37°C for 1 h. Absorbance was then measured at 450 nm using a microplate reader. The cell viability was calculated as the ratio of untreated cells.

### 2.5. Real-Time Qualitative (RT-qRCR) Assays

Target gene transcription levels were determined by RT-qPCR. The cells were seeded in 6-well plates at a density of 2 × 10^5^ cells/well. Stimulation was performed as previously described. Supernatants were harvested and used for enzyme-linked immunosorbent assay (ELISA). The cells were then subjected to RT-qPCR. Total mRNA was obtained using the TRIzol-chloroform method (TRIzol, Kang Century Biotechnology Co., China) and then reverse-transcribed into cDNA using a reverse transcription system (YESEN, Shanghai, China). RT-qPCR was performed to calculate the expression of target gene mRNA. The primer pair sequences are listed in Table 1.

### 2.6. ELISA Assays

The content of ADAM-10 in the supernatants was detected using the ADAM-10 ELISA kit according to the manufacturer's specifications (Fanyin Biotechnology Co., Ltd., Shanghai, China).

### 2.7. Immunofluorescence Assays

RAW264.7 cells were seeded at 3 × 10^4^ cells/confocal dish and treated as described above. After washing, cells were fixed with 4% paraformaldehyde at room temperature for 30 min. Following permeabilization and containment, cells were incubated with primary antibodies (anti-ADAM-10, anti-iNOS, and anti-*β*-tubulin). Next, the cells were incubated with goat anti-rabbit IgG (H+L) FITC-conjugated antibody (S0008, Affinity) or goat anti-mouse IgG (H+L) 594-conjugated antibody (S0005, Affinity). After the final wash, the nuclei were counterstained with 4′,6-diamidino-2-phenylindole (DAPI). Cellular fluorescence was measured using a confocal laser scanning microscope (FV3000, Olympus, Tokyo, Japan).

### 2.8. Flow Cytometry Assays

The cells were harvested by robust pipetting and resuspended in PBS/0.5 bovine serum albumin (BSA)/5 mM EDTA. After fixation with 2% PFA buffer for 15 min at room temperature, the cells were permed with a commercial kit (BioLegend, #426803) according to the manufacturer's protocols and then incubated with either FITC-conjugated ADAM-10 (Biorbyt, #orb8517) or MMP-12 (Bio-Connect, #SC-133151) for 20 min on ice. After washing twice with cold PBS, the cells were analyzed by flow cytometry (Thermal Fisher Attune Nxt).

### 2.9. Statistical Analysis

Data are expressed as the mean ± standard deviation, and SPSS 25.0 and GraphPad Prism 8.0 were used for statistical analysis. *P* < 0.05 was considered statistically significant. NS: no significance; ^∗^*P* < 0.05, ^∗∗^*P* < 0.01, and ^∗∗∗^*P* < 0.001. All statistical graphs and data represent similar results from at least two independent experiments.

## 3. Results

### 3.1. sh ADAM-10 Induces MMP-12 Dysregulation in Macrophages

**Figure 1:**
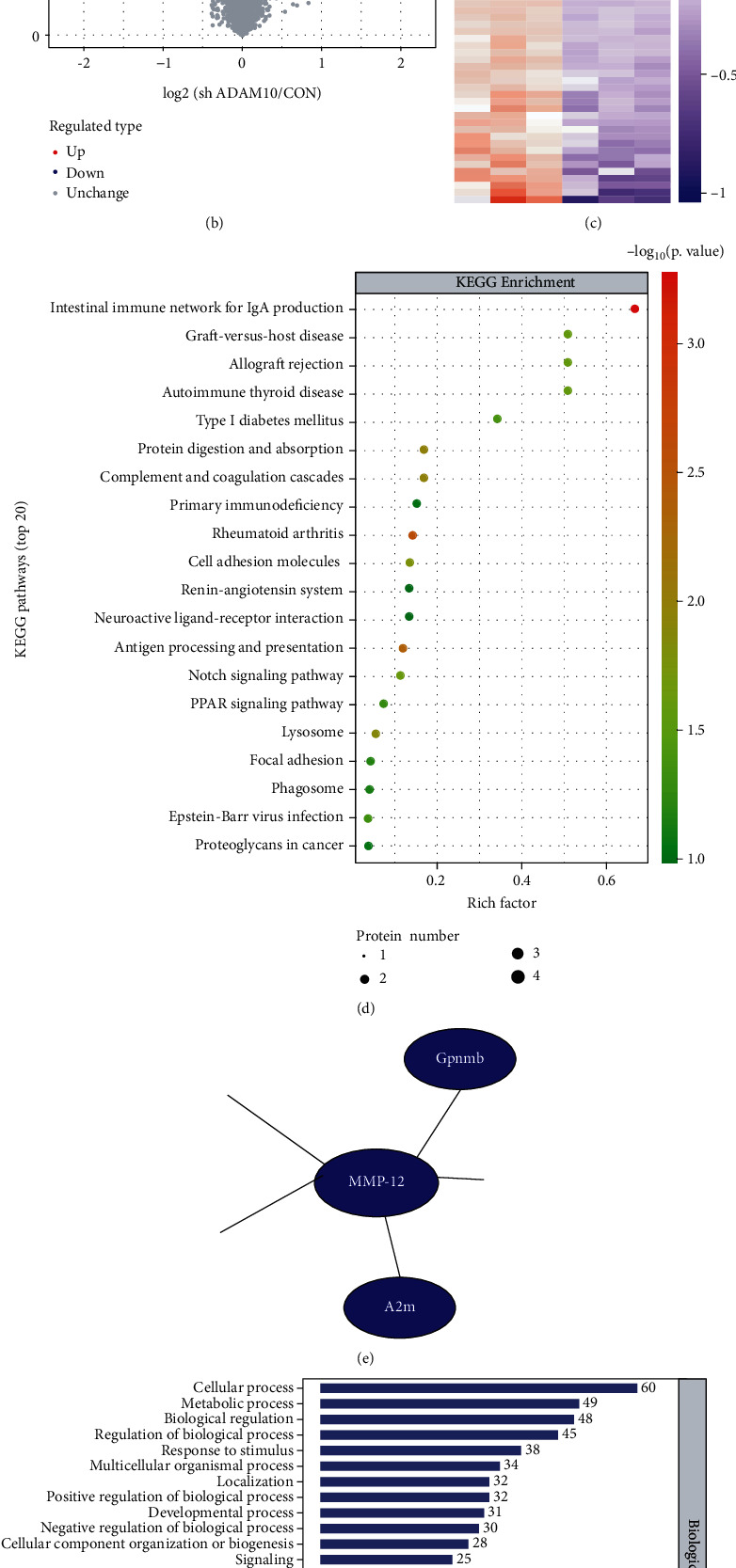
A disintegrin and metalloprotease 10 (ADAM-10) depletion induces matrix metalloproteinase 12 (MMP-12) disorders in RAW264.7 cells. (a) ADAM-10 interference in RAW264.7 cells. Scale bar = 100 *μ*m. The dot plots show the statistical analysis of the expression level of ADAM-10 via qualitative PCR (qPCR). Each dot represents a readout. (b) Tandem mass tag (TMT) results; 40 proteins were downregulated and 30 proteins upregulated in ADAM-10 RAW264.7 depleted cells compared to control cells. Each dot represents a readout. (c) Hierarchical clustering analysis results of the significant proteins from (b). Results are shown in a heat map. (d) KEGG pathway analysis of the different proteins. (e) Protein interaction network analysis revealing that interfered ADAM-10 (sh ADAM-10) decreases MMP-12 expression. (f) Biological Process Gene Ontology (GO) term enrichment analysis results for the different proteins.

We used TMT proteomics to explore the potential protein targets after depletion of ADAM-10. ADAM-10 knockdown significantly reduced the gene expression of ADAM-10 in cells (Figure 1(a)). In TMT experiments, the number of regulated proteins was identified based on the expression fold change (fold change, FC > 1.2 fold normal expression, meaning upregulated by more than 1.2 fold or downregulated by 0.83 fold) and *P* < 0.05. The results showed that 30 proteins were upregulated and 40 proteins were downregulated in sh ADAM-10 cells (vs. the control cells) (Figure 1(b)). Differentially expressed proteins were analyzed using hierarchical clustering. The heat map in Figure 1(c) shows significantly upregulated (red) and downregulated (blue) proteins. These differentially expressed proteins were then parsed and annotated using KEGG pathway analysis, and the number of differentially expressed proteins was counted for specific analysis (Figure 1(d)).  Figure 1(e) shows the 20 most enriched KEGG pathway terms.

Simultaneously, Gene Ontology (GO) classification was performed, and the differential proteins were annotated into three categories: biological process, molecular function, and cellular component (Figure 1(f)). ADAM-10-associated immune response was enriched in terms of biological processes. Furthermore, our protein interaction map showed that ADAM-10 may reduce the protein expression of MMP-12 (Figure 1(e)).

### 3.2. LPS-Induced Macrophage Inflammatory Response Is Modulated by ADAM-10 and MMP-12

**Figure 2:**
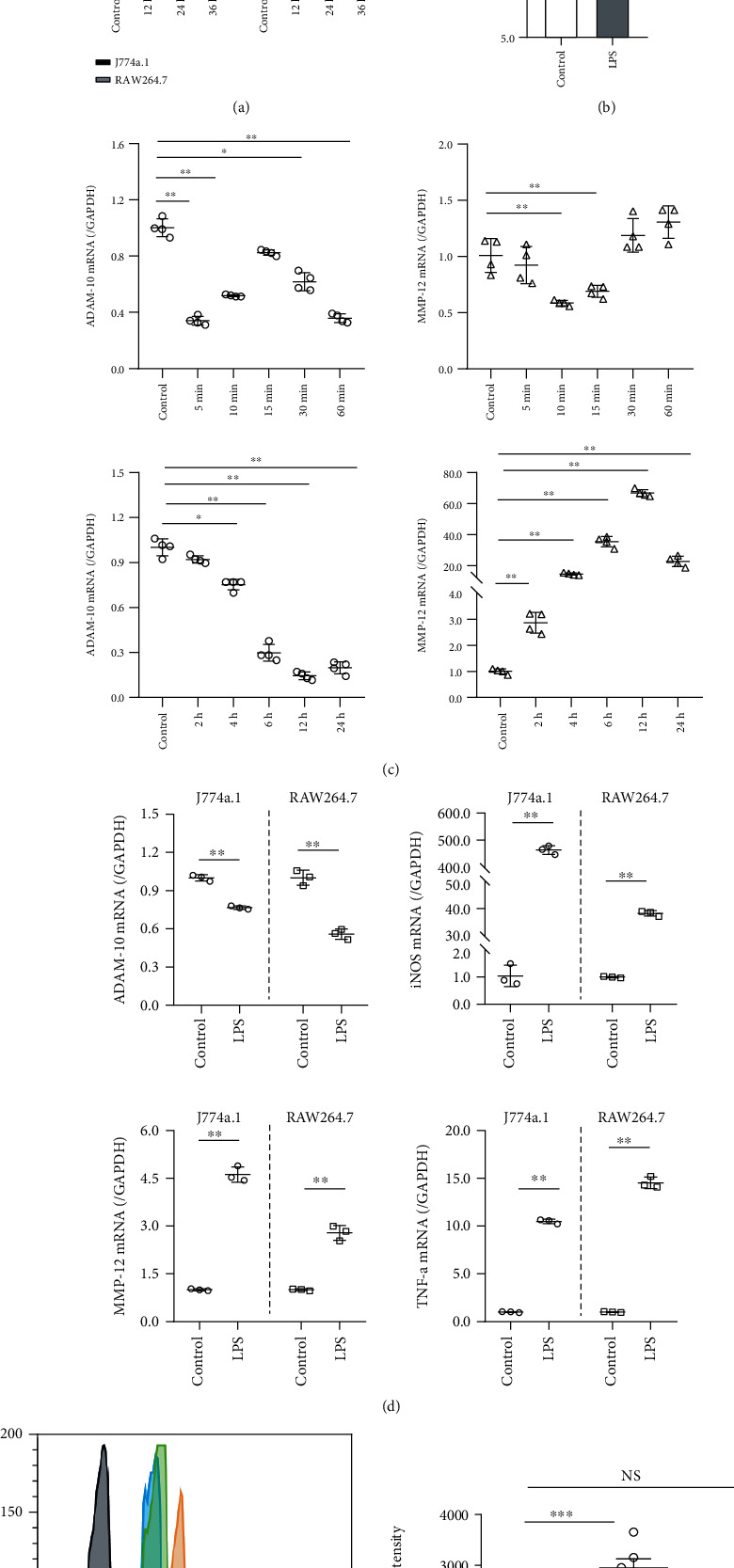
Lipopolysaccharide-promoted proliferation- (LPS-) induced inflammatory responses in macrophages affect A disintegrin and metalloprotease 10 (ADAM-10) and matrix metalloproteinase 12 (MMP-12). (a) The upper bar graph shows increased cell viability induced by LPS; the lower bar graph shows that MMP-12 inhibition had no significant effect on cell viability. Each group contained six samples. (b) The upper dot plot indicates LPS-inhibited ADAM-10 mRNA synthesis, determined by qualitative PCR (qPCR), with each dot representing one read. The lower bar graph shows increased secretion of ADAM-10 proteins by LPS, as determined by enzyme-linked immunosorbent assay (ELISA); each group containing four wells. (c) LPS stimulated ADAM-10 and MMP-12 mRNA synthesis in a time-dependent manner. Each dot represents a readout. (d) The effect of LPS (1 *μ*g/ml, 12 h) on *ADAM-10*, *MMP-12*, *iNOS*, and *TNF-α* transcript levels in J774a.1 and RAW246.7 cells. Each dot represents a readout. (e) Flow cytometry analysis indicated inhibition of MMP-12 which decreased ADAM-10 expression.

In normal J774a.1 and RAW264.7, 1 *μ*g/ml LPS treatment promoted an increase in cell viability for 9–24 h in a time-dependent manner (*P* < 0.05), and 1.5 *μ*M MMP-12 inhibitor did not affect these cells for 12–36 h (Figure 2(a)).

Three different ADAM-10 primer sequences were used to demonstrate the effect of LPS on intracellular *ADAM-10* gene transcription. The results indicated that LPS reduced *ADAM-10* gene expression, and this effect was most prominent with sequences 1 and 3. Sequence 1 was selected for subsequent experiments (Figure 2(b)). The ELISA results showed that LPS increased the content of ADAM-10 in the cell supernatant compared to the CON group (*P* < 0.05, Figure 2(b)).

*ADAM-10* expression was inhibited within 6–24 h of LPS treatment; however, this inhibition was most notable when the cells were exposed to LPS for 12 h. However, *MMP-12* transcription was LPS-promoted and increased the most at 12 h (Figure 2(c)).

The gene expression pattern affected by LPS was subsequently determined in both normal J774a.1 and RAW264. RT-qPCR results showed that LPS had similar roles in the two cell lines. LPS inhibited *ADAM-10* expression and in turn promoted *MMP-12*, *iNOS*, and *TNF-α* expression (Figure 2(d)). Compared with J774a.1 cells, *ADAM-10* transcription was more significantly affected by LPS in RAW264.7 cells. Therefore, RAW264.7 cells were selected for subsequent studies. After LPS stimulation, the protein expression of mature ADAM-10 (ADAM) increased. In contrast, the MMP-12 inhibitor had no significant effect on ADAM-10 (Figure 2(e)).

### 3.3. ADAM-10 Knockdown Reduces LPS-Induced Macrophage Activation by Downregulating MMP-12

**Figure 3:**
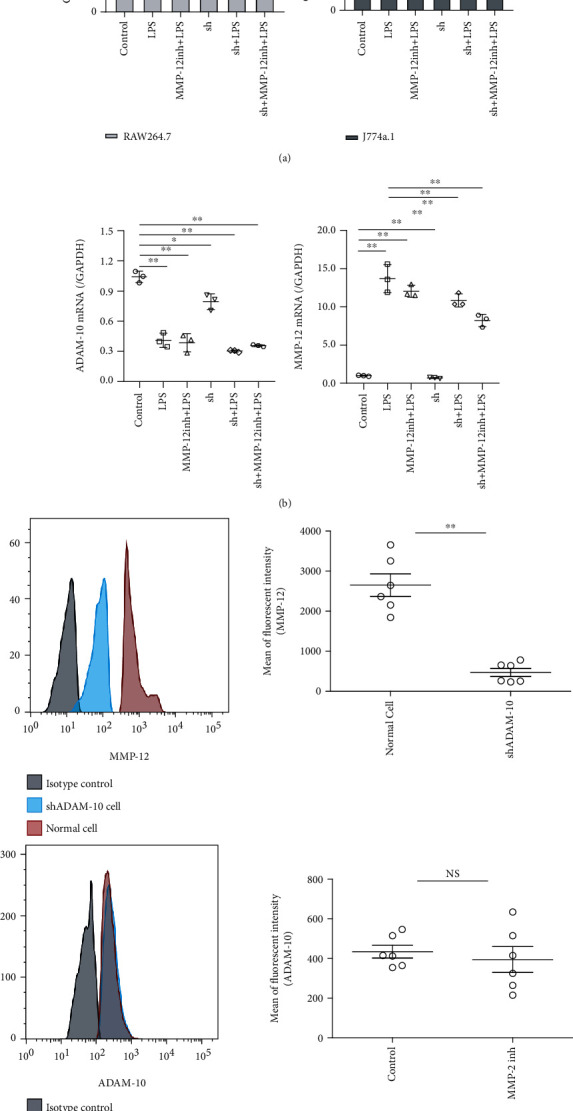
Interfered A disintegrin and metalloprotease 10 (sh ADAM-10) decreases lipopolysaccharide-promoted proliferation- (LPS-) induced macrophage activation via matrix metalloproteinase 12 (MMP-12) downregulation. (a) The effect of LPS and MMP-12 inhibitors on sh ADAM-10 RAW246.7 and sh ADAM-10 J774a.1 cell viability. Each group contained six samples. (b) Real-time qualitative PCR (RT-qPCR) analysis of *ADAM-10* and *MMP-12* transcription levels in response to LPS and MMP-12 inhibitors in sh ADAM-10 RAW246.7 cells. Each group contained six samples. (c) Flow cytometry analysis shows inhibition of ADAM-10 decreased MMP-12 expression, while inhibition of MM-12 has no significant effect on ADAM-10.

Compared to the CON group, LPS treatment for 12 h significantly increased the viability of normal cells (*P* < 0.01). In the MMP-12 inhibitor+LPS group, the MMP-12 inhibitor (12 h) was followed by LPS restimulation for 12 h, and the cell viability of this group was significantly higher than that of the CON group (*P* < 0.01). In sh ADAM-10 RAW264.7 cells, cells were seeded at the same density as RAW264.7 cells; however, the cell viabilities of the sh, sh+LPS, and sh+MMP-12 inh+LPS groups were significantly decreased compared to the CON group (*P* < 0.01). Compared with the LPS group, the cell viability of the sh+LPS and sh+MMP-12 inh+LPS groups also decreased (*P* < 0.05) (Figure 3(a)). The results of our normal and sh ADAM-10 J774a.1 cell viability experiments were similar to our RAW264.7 cell viability experiments, as shown in Figure 3(a).

To determine whether ADAM-10 is an upstream signal, we performed lentiviral transfection in cell culture, in which ADAM-10 was disrupted. Flow cytometric analysis revealed that MMP-12 expression was inhibited. In contrast, inhibition of MMP-12 had no significant effect on the expression of ADAM-10, indicating that ADAM-10 could be an upstream signal and could modulate MMP-12 (Figure 3(c)).

### 3.4. ADAM-10 Overexpression Increases LPS-Induced Macrophage Activation by Promoting MMP-12

**Figure 4:**
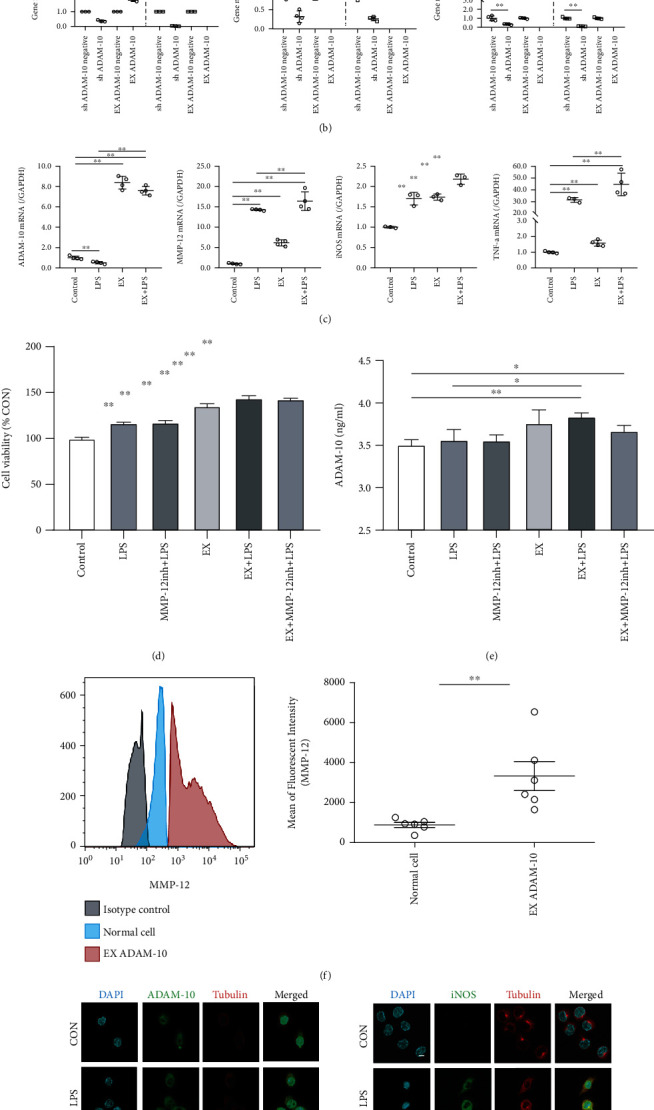
Overexpressing A disintegrin and metalloprotease 10 (EX ADAM-10) increases lipopolysaccharide-promoted proliferation- (LPS-) induced macrophage activation via matrix metalloproteinase 12 (MMP-12) upregulation. (a) Real-time qualitative PRC (RT-qPCR) analysis of the effect of EX ADAM-10 in RAW264.7 cells. Each dot represents a readout. (b) Comparison of the gene transcription levels of interfered ADAM (sh ADAM-10) and EX ADAM-10 in RAW264.7 cells by RT-qPCR. Each dot represents a readout. (c) RT-qPCR analysis of the effect of LPS on gene transcription in EX ADAM-10 RAW246.7 cells. Each dot represents a readout. (d) The effect of LPS and MMP-12 inhibitors on EX ADAM-10 RAW246.7 cell viability. Each group contained six samples. (e) Enzyme-linked immunosorbent assay (ELISA) analysis of ADAM-10 secretion in response to LPS and MMP-12 inhibitors in EX ADAM-10 RAW246.7 cells. Each group contained six samples. (f) Flow cytometric analysis showed that EX ADAM-10 upregulated MMP-12 expression. (g) Immunofluorescence of EX ADAM-10 RAW246.7 cells, followed by treatment with LPS and MMP-12 inhibitors. Scale bar = 10 *μ*m. Data are expressed as the mean ± SD. ^∗^*P* < 0.05 and ^∗∗^*P* < 0.01 compared with the CON group; ^#^*P* < 0.05 and ^##^*P* < 0.01 compared with the LPS group.

ADAM-10 positively regulated MMP-12, iNOS, TNF-*α*, IL-1*β*, and FKN. In other words, the gene expression levels of *MMP-12*, *iNOS*, *TNF-α*, *IL-1β*, and *FKN* in the sh ADAM-10 group were decreased compared with the sh ADAM-10 negative group (*P* < 0.01). Meanwhile, in the EX ADAM-10 group, *MMP-12*, *iNOS*, *TNF-α*, *IL-1β*, and *FKN* gene expression increased compared to the ADAM-10 overexpression negative group (*P* < 0.01) (Figure 4(b)). Following ADAM-10 overexpression, the effect of LPS-induced increase in intracellular MMP-12, iNOS, and TNF-*α* was further increased (Figure 4(c)).

Cell viability was increased in ADAM-10 overexpressing cells (Figure 4(d)), while ADAM-10 protein secretion was increased in the EX+LPS group compared to the CON and LPS groups (*P* < 0.01 and 0.05, respectively) (Figure 4(e)). At the protein expression level, the protein expression levels of MMP-12 were promoted by ADAM-10 overexpression (Figure 4(f)). In the immunofluorescence staining experiments, ADAM-10 had a similar effect as iNOS in terms of expression (Figure 4(g)).

## 4. Discussion

ADAM-10 plays multiple biologically relevant roles in cells. It is widely known that ADAM-10 is involved in the body's inflammatory response [14, 16], but the specific regulatory mechanisms are not clear. In this study, we investigated the effect of LPS on ADAM-10 in macrophages. Simultaneously, by identifying differentially expressed proteins in ADAM-10 depleted cells, we identified the downstream proteins that ADAM-10 may regulate. In addition, using normal, sh ADAM-10, and EX ADAM-10 cells, the regulatory mechanism of ADAM-10 in LPS-induced macrophage inflammatory responses was explored.

MMP-12, also known as matrix metalloproteinase 12 or macrophage elastase, is a member of the matrix metalloproteinase (MMP) family and is regarded as a granulocyte-macrophage proinflammatory marker [17]. It is a phage-colony stimulating factor that induces the expression of inflammatory proteases [18]. MMP-12 is mainly expressed in mature macrophages and exhibits cytolytic properties [19]. In addition, it has been reported in the literature [20] that MMP-12 promotes the recruitment of monocytes/macrophages by inducing the degradation of macrophage extracellular matrix enzymes or/and the production of compounding factors, thereby participating in various inflammatory disease processes. *In vivo* studies [21] found that MMP-12 inhibition reduced the number of F4/80+ macrophages in LPS-stimulated mouse livers. *In vitro* experiments confirmed that MMP-12 expression increased in LPS-stimulated mouse macrophages. Inhibition of MMP-12 can reduce the proliferation of macrophages, so that macrophages are mainly concentrated in the G0/G1 and S phases. Guan et al. [21] confirmed that in LPS-stimulated RAW264.7, MMP-12 regulates various inflammatory cytokines, such as IL-1*β*, IL-6, TNF-*α*, CXCL1, and CXCL3 through the ERK/P38 MAPK signaling pathway, thereby regulating the inflammatory response of macrophages. In addition, MMP-12 is regarded as a neutrophil-macrophage inflammatory marker and proinflammatory protease [18] and is associated with TNF-*α* secretion in macrophages [22].

**Figure 5:**
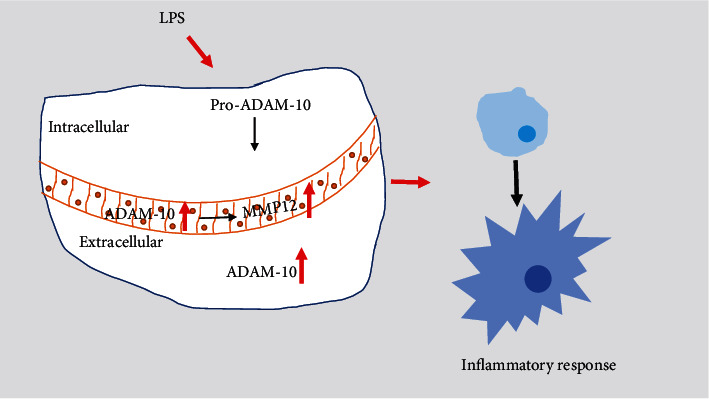
Mechanism of A disintegrin and metalloprotease 10 (ADAM-10) regulation of matrix metalloproteinase 12 (MMP-12) in lipopolysaccharide-promoted proliferation- (LPS-) induced macrophages. LPS promotes macrophage inflammatory responses. Intracellular pro-ADAM-10 is processed into ADAM-10. Additionally, ADAM-10 protein secretion is increased. In this process, ADAM-10 promotes MMP-12 expression, which is jointly related to the LPS-induced inflammatory response.

In normal cells, although multiple ADAM-10 primer sequences were used to examine the effect of LPS on ADAM-10 transcription within macrophages, the RT-qPCR experiment results consistently indicated that LPS reduced the gene expression of ADAM-10 in cells in a time-dependent manner within 4–24 h. Using flow cytometry analysis, we found that LPS promoted the conversion of pro-ADAM-10 to ADAM-10, as well as the extracellular secretion (Figure 5). Along with several literature reports [7, 23, 24], the immature form of ADAM-10 was found to be mainly located in cytoplasmic fibrous structures, whereas the mature form of ADAM-10 mainly adheres to the cell membranes. Therefore, we speculated that LPS promotes the transition from inactive to active ADAM-10 in macrophages and influences the distribution of ADAM-10.

In our positive and negative regulation experiments, where we overexpressed or depleted ADAM-10, the gene expression results showed that ADAM-10 plays a positive role in the gene expression of *MMP-12*, *iNOS*, *TNF-α*, *IL-1β*, *FKN*, and other inflammatory factors. This implies that the overexpression of ADAM-10 promotes the gene expression of these factors, while depletion of ADAM-10 reduces gene expression. ADAM-10 has a notable positive effect on the protein expression of the inflammatory marker iNOS [25, 26]. In sh ADAM-10 cells, the effect of LPS on promoting the iNOS protein expression was reduced, and the effect of the MMP-12 inhibitor also reduced the LPS effect. In EX ADAM-10 cells, the effect of LPS on iNOS protein expression increased, while the effect of the MMP-12 inhibitor on iNOS inhibition was alleviated. This suggests that MMP-12 plays a role in promoting LPS-induced macrophage inflammatory responses. ADAM-10 plays a role in splicing-processing hydrolysis activation in response to TNF-*α* [27]. Both TNF-*α* [28] and iNOS [29] are important proinflammatory factors involved in the inflammatory response. Therefore, we speculated that ADAM-10 positively regulates MMP-12 and participates in LPS-induced macrophage inflammatory responses.

In our cell viability assay, we found that the effect of LPS on cell viability was alleviated in ADAM-10 depletion cells. In ADAM-10 overexpressing cells, the cell viability-promoting effect of LPS increased. Combined with the positive regulation of ADAM-10 and participation in LPS-induced macrophage inflammatory responses, we found that ADAM-10 was involved in LPS-induced macrophage proliferation and activation.

In conclusion, our findings demonstrated the molecular mechanism of ADAM-10 in the proinflammatory response of macrophages to LPS. ADAM-10 regulates the proinflammatory functions of macrophages stimulated with LPS via MMP-12. These experiments have some limitations: first, ADAM-10 depleted cells in this study were transfected with lentivirus, while in ADAM-10 overexpression experiments, the cells were transfected with Lipo 8000 and an inducible plasmid. There are certain differences between these two methods, and it is necessary to further explore why ADAM-10 overexpressing cells cannot be created as stable cell lines. Second, these experiments were designed only to study the effect of various stimuli on the activation of macrophages, and they lacked assessments of the number or proportion of each type of macrophage, whereas subsequent experiments could supplement this. Finally, under the same LPS stimulation conditions, the change in transcriptional trends and in protein expression of the *ADAM-10* gene was not completely unified, and further scientific demonstration is needed. Further *in vivo* experiments are required to confirm the above *in vitro* findings.

## 5. Conclusions

Comprehensively, this study showed that ADAM-10 participates in regulation of macrophages via MMP-10, for example, in the context of LPS-induced inflammation.

## Figures and Tables

**Table 1:** Primer sequences used for gene amplifications.

Gene name	Sequence (5′—3′)
*ADAM-10* (sequence 1)	GTG CCA AAC GAG CAG TCT CA
	GGA AGT GTC CCT CTT CAT TCG
*ADAM-10* (sequence 2)	ATG GTG TTG CCG ACA GTG TTA
	GTT TGG CAC GCT GGT GTT TTT
*ADAM-10* (sequence 3)	TCA TGG GTC TGT CAT TGA TGG A
	TCA AAA ACG GAG TGA TCT GCA C
*MMP-12*	ACC AGA GCC ACA CTA TCC C
	CTC CTG CCT CAC ATC ATA CC
*iNOS*	GGA GCG AGT TGT GGA TTG TC
	CAG CCT CTT GTC TTT GAC CC
*TNF-α*	CGT AGC AAA CCA AG
	GAC AAG GTA CAA CCC ATC GG
*IL-1β*	AAG AGC CCA TCC TCT GTG
	TGT TCA TCT CGG AGC CTG TAG
*FKN*	GTG CCA AAC GAG CAG TCT CA
	GGA AGT GTC CCT CTT CAT TCG

## Data Availability

The data and code generated or analyzed in this study are available from the corresponding authors upon reasonable request.
